# The lived experience of fibromyalgia in female patients, a phenomenological study

**DOI:** 10.1186/2045-709X-19-22

**Published:** 2011-09-19

**Authors:** Francesca Wuytack, Peter Miller

**Affiliations:** 1 Anglo-European College of Chiropractic, 13-15 Parkwood Road, Bournemouth, BH5 2DF, Dorset, UK

## Abstract

**Background:**

Fibromyalgia is a chronic syndrome with no cure. A thorough understanding of the illness experience is therefore key in the palliative care of patients with this condition. In search for supportive treatments fibromyalgia patients often attend a chiropractor or other manual therapist. Knowledge of the meaning and reality of living with this condition to the patient could be considered essential to any health care practitioner playing a role in the management. This study aimed to gain a better understanding of the subjective experience of fibromyalgia, focusing on the personal, occupational and social impact of the condition on patients' lives. This included exploring the patients' views about the future.

**Methods:**

This study employed descriptive phenomenology and adopted Husserl's concept of transcendental subjectivity or "bracketing". This qualitative study involved semi-structured interviews and was undertaken to obtain rich data that reflected the essence of the participants' experience. Participants consisted of six female volunteers, diagnosed with fibromyalgia by the University Hospital Gent, Belgium. Data were analysed using a thematic framework.

**Results:**

Fibromyalgia pervaded all aspects of life. Four main themes arose from data analysis, namely; the impact of fibromyalgia on patients' occupational and personal life, the impact on their future and aspects of social interaction. Nearly all participants had stopped working, giving rise to feelings of uselessness and loss of identity. Leisure activities were also greatly affected. Fibromyalgia was said to alter family bonds, some of which were reinforced, others were broken. The diagnosis was seen as a relief, marking an end to a period of uncertainty. Participants reported ambivalence in interaction. Despite some positive encounters, frustration arising from perceived incomprehension dominated. Consequently patients preferred not to share their experiences.

**Conclusions:**

The study revealed the negative impact of fibromyalgia on patients' lives as comprising of great complexity and individuality. Several implications for health care practitioners can be extrapolated, including the need of a more efficient diagnostic process and increased education about the fibromyalgia experience. Further studies are required to better clarify the multifaceted nature of living with the condition.

## 1. Background

Fibromyalgia is a chronic syndrome of unknown aetiology [1]. No consensus exists regarding its pathophysiology, however, several hypotheses and observations have been made including neuroendocrine, central nervous system and musculoskeletal changes. The most current research has primarily focused on neural plasticity and the process of central sensitization [2]. A recent survey in five Western European countries estimated a life-time prevalence of 2.9% [3], making it a relatively common condition encountered in primary care. Despite efforts to improve the diagnostic process, it remains often a long journey, as it is a diagnosis of exclusion [4].

No cure is currently available and management of the disorder, consisting of a multidisciplinary individualized and symptomatic approach, is unsatisfactory and very challenging for both patients and health care practitioners [5]. Moreover, the prognosis is poor [5]. Pagano et al. [6] confirmed that fibromyalgia is a very disabling condition associated with a very poor quality of life. The most recent EULAR evidence-based recommendations for the management of fibromyalgia reinforce the need for a multifaceted approach [7]. Hence in managing these patients, a thorough understanding of their illness experience is vitally important, and key to achieve the best results [8].

Ritchie and Lewis [9] state that qualitative research, as an independent mode of research enquiry, may be indicated for ill defined and not well understood phenomena to provide a greater understanding of its nature. This reemphasises the value of gaining more iterative data about fibromyalgia. Using the biopsychosocial model as a starting point, in an attempt to better understand and manage the condition, there has been a recent increased interest in qualitative research exploring the complex and multidimensional context of fibromyalgia. Sim and Madden [10], in a metasynthesis of qualitative studies concerned with the illness experience of fibromyalgia, found that themes emerging from previous literature exploring the 'fibromyalgia experience' were multiple, but could be narrowed down to some general main categories; experience of the symptoms, search for a diagnosis, the legitimacy and invisibility of the condition, and coping strategies were the key topics that came forward.

In an attempt to ally with former qualitative studies about fibromyalgia, this study focused on the areas that had had least attention, including a more detailed description of the personal, occupational and social impact of the condition on patients' lives, their views about the future, and issues concerning communication and the expression of feelings.

## 2. Methods

### 2.1 Design

This study employed descriptive phenomenology and adopted Husserl's concept of transcendental subjectivity or "bracketing". This qualitative study involved semi-structured interviews and was undertaken to obtain rich data that reflected the essence of the participants' experience [11].

### 2.2 Participants

The sample of participants consisted of six female patients, officially diagnosed with fibromyalgia by the University Hospital Gent, Belgium, using the American College of Rheumatology 1990 diagnostic criteria. Participants were identified by contacting the self-help group of Gent. Out of the six patients approached, all took part in the study. Their ages ranged from 36 to 66 years (average 51). Participants had been diagnosed for 1 to 9 years and the time between the onset of the symptoms and receiving a diagnosis varied between 1 to 19 years (average 5 years).

### 2.3 Ethical issues

Ethical approval was gained from the Anglo-European College of Chiropractic ethics board in March 2009. Participants were asked to voluntarily take part in the interview. Participants were given the right to withdraw from the study at any point. Numbers instead of names were used during the analysis to assure anonymity and avoid bias.

### 2.4 Data collection

The interviews took place in July 2009 in Belgium, at a location convenient to the participant. The researcher conducting the interviews was not involved in the participants' care to enhance rigour. Two of the six interviews were conducted in hospital (UZ Gent), whereas the other four participants were interviewed at their homes. The participants were asked to complete an information sheet regarding demographic information. The iterative data were collected using semi-structured interviews in which nine questions were asked to all participants, three questions covering various aspects of each aim of this study, illustrated in Appendix 1.

Participants were encouraged to answer freely, allowing them to elaborate on areas of individual importance. Additional questions were asked by the researcher when appropriate. Notes were made by the interviewer concerning non-verbal communication. The interview times varied from fifteen to forty-five minutes and were audio-recorded on mini-disc.

### 2.5 Data analysis

**Figure 1:**
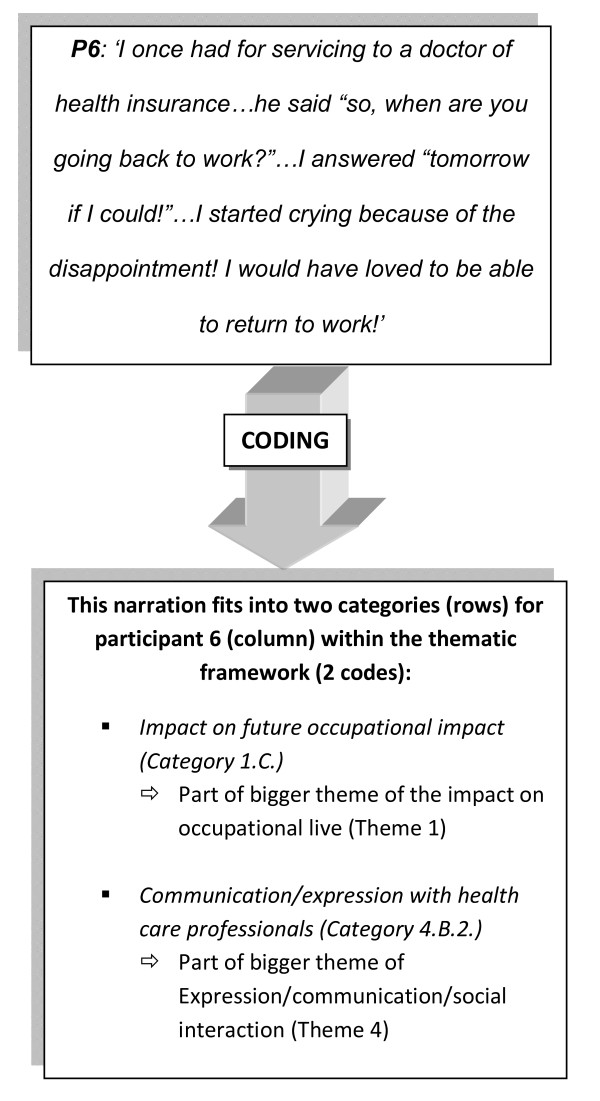
**Example of path of a narration through the data coding process**.

The interviews were transcribed verbatim by the researcher and subsequently rechecked for possible errors to ensure rigour. The original transcripts were translated to English by the researcher. Familiarization of the data took place throughout the process of data collection. Emerging themes and smaller categories were identified by reading the transcript thoroughly multiple times. The transcripts were coded accordingly. An example of the coding process is illustrated in Figure 1. Microsoft Excell^® ^was used to create a table allowing cross-case interpretation for the individual themes and categories; the columns representing the participant number and the rows corresponding to the different themes and categories. The narrations were allocated to one or more theme/category(ies) in the frame according to participant number. To facilitate interpretation of the data, the content of each cell of the Excell^® ^table was synthesized using descriptive keywords, representing the original citation. These descriptions were directly drawn out of the quotes and did not involve interpretation of the content. This was the final stage of the data handling process. Associative analysis was performed by comparing the quotes of all six participants for each single theme/category, identifying patterns and dimensions. The original quotes providing evidence for each statement were labeled and tabulated accordingly. The last stage consisted of a contextual and explanatory analysis.

Findings of the study were discussed in relation to previous research and established concepts. Moreover, personal hypotheses and conclusions were made by the researcher with regard to the research questions. Throughout the data collection and analysis a reflective stance was adopted by the researcher.

## 3. Results & discussion

Four main themes were identified from the data, namely:

• Occupational impact

• Impact on personal life

• Views about the future

• Interaction, communication and expression

### 3.1 Occupational impact

All women in the study had had problems with coping at work since the onset of the fibromyalgia symptoms. The majority stated how they absolutely loved their job, trying to persevere with work despite the complaint and diagnostic uncertainties at that time. Difficulties to continue working were generally related to physical inability and exhaustion. Two participants also mentioned cognitive disturbances as the obstacle for them to keep working. For example participant 5, aged 54 years, said: *'My cognitive disturbances were most annoying...I felt ashamed*...*for forgetting so much.' *Five out of the six participants also mentioned that high stress levels and sometimes an unpleasant atmosphere at work greatly sabotaged their capacity to continue their employment. Work pressure was considered a key barrier to cope. This was very strongly expressed by participant 6 (aged 54): *'There was the factor of stress as well*...*ever increasing demands, I was really overworked and my employer didn't really show much empathy.' *Liedberg and Henriksson [12] related rising working pressure and rationalisation in society to the increased difficulty fibromyalgia patients experienced keeping their job. Previous qualitative studies [13-15] also described an anecdotal link between stressful life events and the onset of the symptoms of fibromyalgia. It is therefore unclear whether occupational pressure experienced by these participants should be considered a trigger or rather a consequence of the condition.

At the time of the interviews five of the six participants were no longer at work, four of which were on benefits and one person on early pension. The exception was participant 3, aged 44, who worked reduced hours and was obliged to continue due to financial difficulties. As the majority of participants were no longer employed, the findings of this study do not coincide with epidemiological studies looking at occupational activity amongst people with fibromyalgia. Henriksson and Liedberg [16], in a survey involving 176 women with fibromyalgia, found that 50% was employed, of which 80% counted to be able to continue working. However, it should be noted that only 15% worked full-time and 58% of the working women had already required a change in work situation. Possibly the type of employment may account for the difference between the working status of the latter study and this study. Several participants in this study worked within the caring sector, which often involves physically demanding activities. Moreover, the variation in national health benefit system and procedures to adapt work situations is likely to be another factor giving rise to this observation, as the study by Henriksson and Liedberg [16] took place in Sweden. However, it could also just be by coincidence resulting from the limited number of participants in this study. The time between the onset of the symptoms and stopping working varied greatly from about six weeks to over ten years. Many participants expressed difficulty in accepting their occupational inability and described it as a long process. This was nicely illustrated by participant 6*: 'Initially when in our counseling group we discussed the way we pictured our lives, my job was still strongly connected with me, but at the end of the year I had taken leave of it...You need to become reconciled with it, but it is a long process to learn and live with that.'*. Despite the love for their job and the struggle to accept their leave, most participants had reconciled themselves to stop working permanently as they perceived a return to their job impossible, nor did they think that appropriate adjustment of their work situation to their physical impairments was practically achievable. This is in line with the findings of Liedberg and Henriksson [12] in a qualitative study looking at the factors important for work disability in patients with fibromyalgia, in which they described the grief many women felt resulting from their lost professional identity. The exception to this, in this study, was a younger person in her thirties. The age difference was most likely one of the factors that made this incapacity even more disruptive and hard to resign to. Lax and Klein [17], looking at the impact of injury or illness on work, emphasized the role of an occupation in an individual's identity and self esteem. Moreover, Arnold et al. [18] stated that the loss of former identity in fibromyalgia patients is strongly linked with loss of job. This observation was supported in this study, for instance participants emphasized that it was a long process to reestablish their identity in life having lost their work role. Together these findings contradict Hallberg and Carlsson's [19] observations of a tendency of fibromyalgia patients to perceive their work situations as unsatisfactory.

There was a general feeling of financial uncertainty, connected with the inability to function at work and being dependant on disability pension. Participants said it was a worrying factor in their lives, due to a sense of fear of being obliged to go back to work again and the constant control and reevaluation of social benefit services. This came across to the participants as a suspicion of lack of credibility: *'I once had to see a doctor for health insurance...he said "so, when are you going back to work?"...I answered "tomorrow if I could!"...I started crying because of the disappointment! I would have loved to be able to return to work!' (Participant 6, aged 54 years)*. Although the way disability benefits are organized is dependent on the health care system of the country, in this case Belgium, the sense of uncertainty about health insurance and subsequent financial worry has come across in previous qualitative studies [18].

### 3.2 Personal life impact

#### Hobbies

In accordance with previous qualitative research involving fibromyalgia patients' life experience, participants reported many different hobbies they could no longer do. From the current understanding of pain perception, it is recognised that distraction, positive mood and emotions have the ability to augment pain inhibition [20]. Interrelating this with the inability of fibromyalgia patients to continue leisure activities, which can be considered as 'pain inhibitors', a downward spiral may be predicted. This may well be the reason why nearly all participants had started new hobbies/activities which they had always wanted to do and which were within their scope. For example participant 6 who proudly said: *'I did manage to start learning Italian. I always wanted to do that but never had the time. I am very glad about that!' *This could be considered a spontaneous action to decrease their suffering, although to them it was merely seen as a way to regain some purpose in life. Hallberg and Carlsson [19] also found in a qualitative study regarding coping with fibromyalgia, that finding distraction in pleasurable activities was one of the coping strategies applied.

A great variety of symptoms have been reported as part of fibromyalgia syndrome in the current literature [21]. Participants in this study gave an account of the numerous ways their hobbies were affected by specific complaints. Pain and stiffness interfered with both gross exercise like dancing, and fine motor activities like painting and knitting. Fatigue impeded travelling and fitness training while cognitive disturbances were said to cause trouble reading books. The latter was directly mentioned by participant 4, aged 54*: 'I love reading! I always hoped there would be a time when I could read more books, but with the concentration disturbances I can't. Leafing through magazines is all I can manage.' *The multitude of symptoms interfering with daily life emphasises the importance of looking at the condition as a complex disorder rather than just a pain syndrome. Furthermore, the constancy yet unpredictability of the disorder was regarded as a major source of having to refrain from previous pursuits. This is in line with Arnold et al. [18] and Cunningham and Jillings [22] who both observed the 'all round' restrictive effect of the ubiquitousness of the symptoms.

Hellstrom et al. [23], in a phenomenological study of fibromyalgia, contributed this cessation of activities to the notion that 'as long as one has not tried to do something one finds desirable, the hope of being able to do it is still intact'. However, in this study the fear to resume activities reported by some participants was not solely based on the assumption of inability, rather it resulted from actual previous experience of failure to do so. One participant stated that she had to cease sewing because of hand cramps and how she now doesn't dare to restart out of fear not being able to do it again.

#### Family life

The importance of family support, both practically and emotionally, was emphasised by all participants. By some it was expressed as fortunate to have help, by others it was the lack of it that made them realise its value. The husband, as the most intimate relation, was said to have the greatest assisting role. For that reason, feelings of gratitude for this empathy were abundantly articulated by the participants as they recognized the challenge their condition had put on their marriages. For example, one participant strongly claimed (age 66)*: 'The support of my husband is worth gold!' *Alternatively for some participants the onset of fibromyalgia signified an end to their relationship which they contributed to a lack of understanding from their partner. The impact of one's partner was commented on by participant 4*: 'My husband at the time my symptoms started showed really no understanding whatsoever...we divorced...The greatest change for me now is a very loving partner. He deals with my fibromyalgia so well...Yes, that is my best gift.'*

All women in this study had children, which were said to also play an important part in providing a supportive environment. However, the participants with children under ten years of age expressed that their children were not old enough to understand their mother's situation. Moreover, the constant care of young children was hard to take.

Similar to findings in other research [18], this study found feelings of guilt were experienced by women. Women felt guilty that their husbands had to undertake more household responsibilities and felt guilty that they were unable to give their children the attention they deserved. Participant 4 commented *'My youngest son was a teenager at the time my symptoms started and he had troubles of his own...that made it harder on him I think, because I couldn't always be there for him.' *In a metasynthesis of qualitative research about experiencing fibromyalgia, similar conclusions of the strain on family relationships were drawn [[10], citing [24,25,12]]. Cunningham and Jillings [22] also related this to the additional burdens for family members resulting from such disability, while Hallberg and Carlsson [19] described it as a role change within the family. Moreover, Hellstrom et al. [23] observed how this resulted in experiencing feelings of insufficiency and uselessness in the patients in their phenomenological study of fibromyalgia. In this study, this sense of inability was most intense with participant 3 who was still working, having to prioritize her job above her family in order to endure it. Family members working within health care were in some cases of great assistance, but in other cases skepticism from them was considered even more hope breaking. The following quote from participant 5 exemplifies the latter: *'My brother in law is a GP, he was the one who told me I had fibromyalgia most likely, but he also said he did not want to have me as his patient anymore because he wouldn't be able to help me anyway...That was a bit of a slap in my face.'*

Looking at these findings from the perspective of a health care professional considering management and coping strategies for fibromyalgia patients, attention must be paid to the importance of family support and understanding, an issue which is currently scarcely addressed (in Belgium). Educational programs concerning fibromyalgia syndrome for families of patients would be a further step and could be of great value in the care of these patients. Moreover, extra household help would also significantly diminish the stress put upon these families.

#### Identity/personality/acceptance

In health psychology, the disruptive impact of chronic illness on personal identity has been extensively studied [26]. Bury in 1982 [27] put forward the concept of 'biographical disruption' resulting from illness. He described this process in three stages consisting of the disruption of taken-for-granted assumptions, rethinking of biography and self-concept, and finally the mobilisation of resources. In accordance with these general principles about chronic illness and with previous qualitative research about fibromyalgia [14,15,28], the women in this study communicated an existential breakdown arising from their life-sapping disabilities. This identity collapse was illustrated by participant 1 (aged 66): *'I used to be a real chatterer...when my complaints started I hardly said anything for nearly eight years.' *Restructuring their identity was considered a struggle but crucial to regain contentment in life. The intricacy of this process was commented on by participant 5 (aged 54): *'It took me two years to learn to accept it and learn to hold up...you can't understand...I had been so healthy and quiet suddenly I had all these symptoms I had to learn to accept...And then little by little I regained some hope, but it took a long time.'*

### 3.3 View about the future

The data from this study demonstrates a clear evolution in the participants' views of their future. This continuum of changing prospects is closely related to receiving a diagnosis. Before being given the label of fibromyalgia, they all reported a long search characterized by uncertainty and consequent worry about the nature of their complaints as well as their future. This was particularly strongly expressed by participant 6: '*It takes ages before they tell you what you have! You have been through a lot of tortures, all tortures! It really is a grisly time!' *Similar experiences of fear and frustration stemmed from a qualitative study by Raymond and Brown [15]. The time between the onset of the symptoms and being diagnosed ranged from one to nineteen years. During this time, many participants testified being commonly confronted with disbelief. As stated by participant 1*: 'They often said it was sitting between my ears!'*

Receiving a label and entering the sick role is wanted by patients both for their own understanding as well as a key for social acceptance [29]. Similar to what prior qualitative research observed [19,23,30,31], the diagnosis created a sense of relief and reassurance. This was nicely described by participant 4*: 'I was happy when I got the diagnosis; finally the child was given a name.' *However, for some these feelings were quickly replaced by new questions and anxieties. While for others it gave them enough ground to start exploring coping strategies. The participants had been diagnosed from anywhere between one and nine years. Generally their future life picture had been altered and accepted to one adjusted to their condition. However, for some participants this was said to be beyond their abilities due to their life circumstances. Especially the younger women still refused to be at peace with this new outlook; however, it could also be related to the fact that they were the ones that had been diagnosed most recently. Moreover, some participants expressed feelings of uncertainty about the evolution of their complaints. Participant 3 stated this directly*: 'I now see my future as very uncertain actually...you really don't know how it is going to evolve.'*

Key factors said to be responsible for a progress to more satisfactory but modified future hopes, were personal strength and perseverance. These were considered paramount in the process of acceptance, which was in turn expressed to be the major step in this 'future shift'. Further, several studies [13,18,19,22] including this one revealed that the strategy of pacing and adapting their demands to their own resources was mainly used to manage. As participant 6 openly avowed*: 'You have to resign yourself to it to learn and live with it.' *This shows the ambiguity with which the participants had to deal daily. Mengshoel and Heggen in 2004 [30] explored the recovery from fibromyalgia in a sample of five women and observed a similar paradox; the resistance to play the sick role gave them strength to improve, but they recovered by reducing the mismatch between their abilities and obligations. However, Hallberg and Carlsson [19] noted that within their interview study, patients expressed difficulty in redefining new bounds for their capability, which was also apparent from this study's transcripts. The women in this study said that the unpredictability of their symptoms obliged them to live on a day-to-day basis making it difficult to plan, as one participant put it*: 'It is like a phantom, it jumps from one area to another, so you constantly have to change the way you handle your body.' *Nevertheless the majority was not pessimistic at all about their future, which contradicts results of other studies [19,23]. These findings also oppose the assumptions made by Hellstrom et al. [23] that patients unintentionally take advantage of a little understood unpredictable illness. Other factors, evident from the data, that aid the process of adapting one's life expectations and then resigning to them, were family support, self help groups and alternative medicine.

### 3.4 Expression, interaction and communication

#### Emotional aspect of interaction

The emotional experience of their illness was greatly influenced by interaction with others. Reduced social contact gave rise to feelings of sadness and loneliness. Participant 5 commented*: 'When I was sitting at home alone all the time, it was as if the walls were coming at me...I felt depressed.' *Sim and Madden in 2008 [10] concluded in their metasysthesis that this loss of social activity emanated from the need to establish priorities. However, in this study the loss of some friends resulted more from a lack of understanding and social acceptance of fibromyalgia. Participants 5 described how friendships were lost: *'I do have to say that many friends stayed behind when I got fibromyalgia, but I comfort myself that these weren't true friends.' *This was also apparent from other research [18]. Despite this sense of regret, the women in this study said they often felt frustrated and hopeless when interacting with others; feelings that were mainly provoked by the invisibility of their illness and the difficulty of explaining their condition, which has been well documented [13,19,22,23,31]. This frustration, felt by all participants, was directly communicated by participant 3: *'And people saying "You are looking well."...They don't understand it because they don't see it.' *Inquires from family and closer friends were received more happily, although most participants disliked being asked too many questions about their health.

Self-help groups are considered to have a valuable role in health care in general [32]. Support from other fibromyalgia patients appears to be an important factor in freeing them from the feeling of isolation. The value of mutual understanding amongst fibromyalgia patients was commented on by participant 3*: '...That is the nice thing about meeting in a self-help group, you don't have to explain and justify yourself all the time.' *The findings of this study, however, did not distinguish between helpful and unhelpful forms of support as was concluded by Cunningham and Jillings [22]. Moreover, all participants were members of a self-help group, hence, the perceived importance of such groups in this study may not represent fibromyalgia patients' opinion in general.

A greater need of appreciation was also expressed by many participants, which may indicate increased emotional vulnerability. Particularly participant 4 stressed the importance of it: *'It really feels wonderful when people tell me they like my work, that is something to hold on to, in a way that is a support...Everybody needs appreciation, but those who are ill need it even more.'*

#### Expression and communication

Looking at the communication of their experiences to others, there was a tendency of not wanting to talk about it. As participant 4 frankly said*: 'Actually I don't talk about it with anyone. If they ask me how I feel, I systematically reply "As good as it can be." That is my standard answer, and it is true.' *This holding back of communicating how they felt was said to result from the incomprehension with which they were confronted, the struggle to describe it and the desire to avoid unwanted reactions. All participants stated that this loss of confidence that people would understand was based on their own experience. Participant 3 commented*: 'I used to give a lot of explanation, but now I can't be bothered anymore. There are very few people who understand it.' *On the other hand the lack of expression may be related to the fact that focusing on symptoms such as pain will increase its levels [20]. Patients therefore may intuitively want to avoid bringing it to the foreground. But this is paradoxical; participants did feel the need to open their heart to someone they confided in from time to time. However, this want of conversation on the topic of their suffering seemed to emanate from a general environment of incredulity rather than the desire to discuss it. In other words, perhaps they wouldn't wish to converse about it, were it not for the experienced all-round incomprehension. Thus it can be concluded, that a major future challenge for the health care profession is to increase awareness about the condition, as it cannot be expected to be the task of the patients because they find themselves in too vulnerable a situation to do so. In agreement with Raymond and Brown [15] who observed a silence out of fear of rejection, participants in this study said they wanted to protect themselves against undesirable reactions by keeping everything to themselves.

In a recent study [31], looking at patient's experiences of the process of getting the diagnosis of fibromyalgia, they found that women suffering from fibromyalgia continued to experience stigmatisation after being diagnosed. The results of this study also demonstrated that the diagnosis merely meant an existential reassurance for themselves, rather than liberation from social skepticism. The participants all recollected too many encounters with disbelief and incomprehension. This study also confirmed previous research [18,19,22,30] that fibromyalgia patients feel they are often viewed as malingerers. Participant 2 reinforced the difficulty of gaining recognition from others: *'They don't feel what you feel and it is so hard to tell them what you feel...If you break your arm you can see it, but not in the case of fibromyalgia.' *Some participants, however, did seem to notice a slight positive change over time in the way fibromyalgia patients are looked upon, however, this could be an anecdotal coincidence.

#### Interaction with health care practitioners

On their journey through many health care services, all the women had experienced disbelief, denial and lack of commitment and interest. Participant 4 had an experience which was typical of the latter: *'At the start of my complaints I visited a neurologist who had frankly said: "There are believers and non-believers and I am a non-believer, so we're not going to talk about that." That really closed a door for me.' *Most said to have had so many negative encounters, that they were utterly grateful to those health practitioners who had shown respect and understanding. This is in line with findings of prior research [19,22,31]. Of interest for health care professionals are the elements that created a positive encounter from the participants' perspective. Respect, belief, guidance, commitment and realistic opinions were the aspects that were valued mostly. Similarly Cunningham and Jillings [22] concluded from their interview study that a collaborative relationship with health care providers would form a basis for comprehensive, supportive care and help to address the complexities of symptom management.

Hellstrom et al. in 1998 [14] did a phenomenological study looking at doctors' attitudes towards fibromyalgia. They found that doctors tended to dislike clinical situations in which they did not feel in control and that the diagnosis also relieved them of a feeling of inability. Moreover, doctors were inclined to focus on symptoms that could be managed within a biomedical setting; however, they stressed the importance of good communication and showing empathy. Looking at this other side of the picture, it is interesting to note that uncertainty and attempts to pain management are issues that not only the patients have to deal with. Moreover, agreement is present on the need of good interaction. However, it should be noted that the doctors from that study were volunteers, showing already a certain interest in the matter.

## 4. Conclusion

The use of a phenomenological design allowed the researcher to gather rich, iterative data and was considered the best choice for trying to gain an inside in the participants' experience. The findings from this study confirm the life disruption caused by fibromyalgia, obliging patients to reform their family life, occupational and social identity. This took place in an atmosphere of uncertainty, firstly about their diagnosis and afterwards about the management and future. This data would suggest that efforts to speed up the diagnostic process would significantly reduce the stress of uncertainty patients experience, which in turn might slow down the onset of complaints. The results also clearly indicated many existing communication barriers and dissonance. Educating the general public and health care professionals about the experience of fibromyalgia may well resolve many of these difficulties, as lack of understanding and empathy was said to be the major block during interaction. Further research exploring the experience of doctors, the family of patients, as well as the general public would be useful to compare their perceptions and develop strategies to increase harmony in communication. Also the observed duality existing in the desire to express and communicate their complaints is a field which requires investigation. There was a consensus between patients who thought they coped well, that balance is the key to proper management. Where this balance lies is individual, which may explain the existing controversies concerning the syndrome. Only six patients were interviewed due to time restraints and data saturation may not have been achieved. Hence studies involving larger samples would also be valuable to try and identify any subgroups or typologies. Although many parallels were discerned between the participants' illness experiences, the qualitative design and the individuality of the syndrome do not allow to truly generalize the findings. All participants came from a same ethnic background, however, the study did not aim to identify ethnic difference in the fibromyalgia experience. Participants were all member of a self-help group which may be a select group rather than a true sample of fibromyalgia patients. The researcher tried to avoid influence of own preconceptions in the analysis. Ideally quality control by other researchers should have taken place [33], but this was not practically achievable. Concerning the interview setting, a decreased interview time and openness observed with those interviewed in hospital, even though privacy was assured. The translation of the transcript was done by the researcher who has mastered both the Dutch and English language. Moreover, the meaning of the transcript was preserved in the translation.

## 5. List of abbreviations

EULAR: European League Against Rheumatism.

## 6. Competing interests

The authors declare that they have no competing interests.

## 7. Authors' contributions

FW designed the research, carried out the data collection and analysis and wrote the manuscript. This study was done as a research thesis undertaken by FW in completion of a Master in Chiropractic. PM assisted in the design of the research and preparation of the manuscript. Both authors read and approved the final manuscript.

## 8. Appendix 1: Questions of the semi-structured interviews

*Impact on personal, social and occupational life*

▪ How has fibromyalgia influenced your professional life/career?

▪ What are your preferred leisure activities? What impact did your condition have on them?

▪ How does your partner/husband/wife/housemate cope wilt it? Do you feel they understand and except it? How did and do they respond?

*Patients' views, attitudes and behaviours with regards to their future*

▪ When you were first diagnosed, how did you see your future?

▪ How do you see your future now?

▪ If changed: what do you think brought about this change?

*Patients' views, attitudes and behaviours with regards to their future*

▪ When you were first diagnosed, how did you see your future?

▪ How do you see your future now?

▪ If changed: what do you think brought about this change?

*How do patients with fibromyalgia feel about expressing what they experience? And how do they feel they are perceived by others?*

▪ How do you feel when people ask you 'How are you'? When close friend/family ask it? When more distant relations ask it?

▪ Do you feel you can say how you feel to close family/friend/doctors?

▪ How do you perceive their reactions if you do express how you feel?

## References

[B1] Nampiaparampil DE Shmerling RH A review of fibromyalgia Am J Manag Care 2004 10 11 794 800 15623268

[B2] Jensen KB Kosek E Petzke F Carvill S Fransson P Marcus H Williams SC Chov E Giesecke T Mainguy Y Gracely R Ingvar M Evidence of dysfunctional pain inhibition in Fibromyalgia reflected in rACC during provoked pain Pain 2009 144 1-2 95 100 10.1016/j.pain.2009.03.018 19410366

[B3] Branco JC Bannwarth B Failde I Abello Carbonell J Blotman F Spaeth M Saraiva F Nacci F Thomas E Caubere JP Le Lay K Taieb C Matucci-Cerinic M Prevalence of fibromyalgia: a survey in five European countries Seminars in Arthritis and Rheumatology, Article in press 2009 http://www.sciencedirect.com/[online] [cited 2010 Jan 10] 10.1016/j.semarthrit.2008.12.003 19250656

[B4] Eich W Häuser W Friedel E Klement A Herrmann M Petzke F Offenbächer M Schiltenwolf M Sommer C Tölle T Henningsen P Definition, classification and diagnosis of fibromyalgia syndrome Schmerz 2008 22 3 255 66 10.1007/s00482-008-0671-7 18478271

[B5] Sumpton JE Moulin DE Fibromyalgia: presentation and management with a focus on pharmacological treatment Pain Res Manag 2008 13 6 477 83 19225604 10.1155/2008/959036 PMC2799316

[B6] Pagano T Matsutani LA Ferreira EA Marques AP Pereira CA Assessment of anxiety and quality of life in fibromyalgia patients Sao Paulo Med J 2004 122 6 252 8 15692719 10.1590/S1516-31802004000600005 PMC11126175

[B7] Carville SF Arendt-Nielsen S Bliddal H Blotman F Branco JC Buskila D Da Silva JAP Dannekiold-Samsoe B Dincer F Henriksson C Henriksson KG Kosek E Longley K McCarthy GM Perrot S Puszczewicz M Sarzi-Putinni P Silman A Spath M Choy AH EULAR evidence-based recommendations for the management of fibromyalgia syndrome Ann Rheum Dis 2008 67 4 536 541 17644548 10.1136/ard.2007.071522

[B8] Huynh CN Yanni LM Morgan LA Fibromyalgia: diagnosis and management for the primary healthcare provider Journal of Womens Health 2008 17 8 1379 87 10.1089/jwh.2007.0656 18788986

[B9] Ritchie J Lewis J Ritchie J, Lewis J The functions of qualitative research Qualitative Research Practice, a guide for Social Science students and Researchers 2003 1 London, Thousand Oaks, New Delhi: SAGE publications 26 34

[B10] Sim J Madden S Illness experience in fibromyalgia syndrome: A metasynthesis of qualitative studies Soc Sci Med 2008 67 1 57 67 10.1016/j.socscimed.2008.03.003 18423826

[B11] Byrne MM Understanding life experiences through a phenomenological approach to research AORN J 2001 73 4 830 832 10.1016/S0001-2092(06)61812-7 11303472

[B12] Liedberg GM Henriksson CM Factors of importance for work disability in women with fibromyalgia: an interview study Arthritis Rheum 2002 47 3 266 274 10.1002/art.10454 12115156

[B13] Hallberg LRM Carlsson SG Coping with fibromyalgia Scand J Caring Sci 2000 14 1 29 36 12035259 10.1111/j.1471-6712.2000.tb00558.x

[B14] Hellstrom O Bullington J Karlsson G Lindqvist P Mattsson B Doctors' attitudes to fibromyalgia: a phenomenological study Scand J Soc Med 1998 26 3 232 237 9768454 10.1177/14034948980260030201

[B15] Raymond MC Brown JB Experience of fibromyalgia Canadian Family Physician 2000 46 5 1100 1106 10845136 PMC2144885

[B16] Henriksson C Liedberg G Factors of importance for work disability in women with fibromyalgia J Rheumatol 2000 27 5 1271 1276 10813300

[B17] Lax MB Klein R More than meets the eye: social, economic and emotional impacts of work-related injury and illness New Solut 2008 18 3 343 360 10.2190/NS.18.3.i 19058415

[B18] Arnold LM Crofford LJ Mease PJ Burgess SM Palmer SC Abetz L Martin SA Patient perspectives on the impact of fibromyalgia Pat Educ Couns 2008 73 1 114 120 10.1016/j.pec.2008.06.005 PMC2564867 18640807

[B19] Hallberg LRM Carlsson SG Psychosocial vulnerability and maintaining forces related to fibromyalgia Scand J Caring Sci 1998 12 2 95 103 10.1080/02839319850163020 9801630

[B20] Craig KD McMahon SB, Koltzenburg M Emotions and psychobiology Wall and Melzack's Textbook of Pain 2006 5 China: Elsevier Churchill Livingstone 231 239

[B21] Brady DM Schneider MJ Fibromyalgia syndrome: a new paradigm for differential diagnosis and treatment J Manipulative Physiol Ther 2001 24 8 529 541 10.1067/mmt.2001.118202 11677554

[B22] Cunningham MM Jillings C Individuals' descriptions of living with fibromyalgia Clin Nurs Res 2006 15 4 258 273 10.1177/1054773806291853 17056769

[B23] Hellstrom O Bullington J Karlsson G Lindqvist P Mattsson B Phenomenological study of fibromyalgia, patient perspectives Scand J Prim Health Care 1999 17 1 11 16 10.1080/028134399750002827 10229986

[B24] Cudney SA Butler MR Weinert C Sullivan T Ten rural women living with fibromyalgia tell it like it is Holist Nurs Pract 2000 16 3 35 45 10.1097/00004650-200204000-00009 11913226

[B25] Henriksson CM Living with continuous muscular pain - patient perspectives Scand J Caring Sci 1995 9 2 67 86 7617994 10.1111/j.1471-6712.1995.tb00390.x

[B26] Selss D Sledge WH Wieland M Walden D Flanagan E Miller R Davidson L Cascading crises, resilience, and social support within the onset and development of multiple chronic conditions Chronic Illn 2009 5 2 92 102 10.1177/1742395309104166 19474232

[B27] Bury M Chronic illnesses biographical disruption Sociol Health Illn 1982 4 2 167 182 10.1111/1467-9566.ep11339939 10260456

[B28] Raheim M Haland W Lived experience of chronic pain and fibromyalgia: Women's stories from daily life Qual Health Res 2006 16 6 741 761 10.1177/1049732306288521 16760533

[B29] Miczo N Stressors and social support perceptions predict illness attitudes and care-seeking intentions: re-examining the sick role Health Commun 2004 16 3 347 361 10.1207/S15327027HC1603_5 15265755

[B30] Mengshoel AM Heggen K Recovery from fibromyalgia - previous patients' own experiences Disabil Rehabil 2004 26 1 46 53 10.1080/09638280410001645085 14660198

[B31] Undeland M Malterud K The fibromyalgia diagnosis-hardly helpful for the patients? Scand J Prim Health Care 2007 25 4 250 255 10.1080/02813430701706568 18041660 PMC3379768

[B32] Kogstad OA Self-help groups are effective Tidsskr Nors Laegeforen 2009 129 1 40. 10.4045/tidsskr.2009.0409 19119299

[B33] Greenhalgh T Taylor R Papers that go beyond numbers (qualitative research) BMJ 1997 315 7110 740 743 9314762 10.1136/bmj.315.7110.740 PMC2127518

